# Role of cytarabine in paediatric acute promyelocytic leukemia treated with the combination of all-trans retinoic acid and arsenic trioxide: a randomized controlled trial

**DOI:** 10.1186/s12885-018-4280-2

**Published:** 2018-04-03

**Authors:** Li Zhang, Yao Zou, Yumei Chen, Ye Guo, Wenyu Yang, Xiaojuan Chen, Shuchun Wang, Xiaoming Liu, Min Ruan, Jiayuan Zhang, Tianfeng Liu, Fang Liu, Benquan Qi, Wenbin An, Yuanyuan Ren, Lixian Chang, Xiaofan Zhu

**Affiliations:** State Key Laboratory of Experimental Hematology, Department of Paediatrics Haematology, Institute of Hematology and Blood Diseases Hospital, Chinese Academy of Medical Sciences and Peking Union Medical College, 288 Nanjing Road, Tianjin, 300020 People’s Republic of China

**Keywords:** Acute promyelocytic leukaemia, All-trans retinoic acid, Arsenic trioxide, Paediatric, Cytarabine

## Abstract

**Background:**

The combination of all-trans-retinoic acid (ATRA) and arsenic trioxide (ATO) has been suggested to be safe and effective for adult acute promyelocytic leukaemia (APL). As of 2010, the role of cytarabine (Ara-C) in APL was controversial. The aim of this study was to test the efficacy and safety of ATRA and ATO in paediatric APL patients. Also, we assessed whether Ara-C could be omitted in ATO and ATRA- based trials in children.

**Methods:**

We performed a randomized controlled trial in paediatric APL patients (≤14 years of age) in our hospital from May 2010 to December 2016. All of the patients were assigned to receive ATRA plus ATO for induction followed by one course of idarubicin (IDA) and ATO (28 days). The patients were then randomly assigned to receive two courses of daunorubicin (DNR, no- Ara-C group) or DNR + Ara-C (Ara-C group). All of the patients were followed with maintenance therapy with oral ATRA, 6-mercaptopurine, and methotrexate for 1.5 years.

**Results:**

Among the 66 patients, 43 were male and 23 were female. All of the patients achieved complete remission (CR) with the exception of one who gave up the treatment. During induction therapy, all toxicity events were reversed after appropriate management. Thirty patients in the Ara-C group underwent 57 courses of treatment, and 35 patients in the no-Ara-C group underwent 73 courses of treatment. No significant differences in age, genders, white blood cell counts, haemoglobin levels, and platelet counts were found between the Ara-C and no-Ara-c groups. Greater myelosuppression and sepsis were observed in the Ara-C group during the consolidation courses. No patient died at consolidation, and only one patient relapsed. No differences were found in event-free survival, disease-free survival and overall survival between the two groups. Additionally, our analysis of the arsenic levels in the plasma, urine, hair and nails of the patients indicated that no significant accumulation of arsenic occurred after ATO was discontinued for 12 months.

**Conclusions:**

Overall, ATO and ATRA are safe and effective for paediatric APL patients and Ara-C could be omitted when ATO is used for two courses.

**Trial registration:**

ClinicalTrials.gov (NCT01191541, retrospectively registered on 18 August 2010).

## Background

All-trans retinoic acid (ATRA) and anthracycline-based chemotherapy is highly effective for newly diagnosed cases of acute promyelocytic leukaemia (APL) [1, 2]. Additionally, arsenic trioxide (ATO) is the most potent single agent in APL therapy [3, 4]. Furthermore, the combination of ATRA and ATO has been suggested to be safe and effective as a frontline treatment, at least in adult patients with low- and intermediate- risk disease [5–11]. In paediatric APL, the use of ATO and ATRA as an induction and consolidation chemotherapy regimen has also resulted in excellent outcomes and improved the long-term prognosis [12, 13]. Our retrospective analysis also indicated that using a combination including ATRA and ATO resulted in good therapeutic outcomes in children with APL [14].

In the pre- ATO era, the role of cytarabine (Ara-C) in APL was controversial [15–17]. More recently, the introduction of ATO and its use in association with ATRA, either with or without chemotherapy, has further improved patient outcomes by allowing the intensity of chemotherapy to be minimized while maintaining a high level of anti-leukaemic efficacy [7, 11, 18]. However, when our trial began, the feasibility of treating patients with APL without chemotherapy was unknown. Furthermore, whether the use of the combination of ATO and ATRA would allow Ara-C to be omitted in consolidation chemotherapy has not been a prospectively studied in children.

Here, we present the results of the protocol-specified analysis of China children with APL study 2010 (CCAPL2010). We assessed whether a combination including ATRA and ATO is safe and effective in paediatric APL. Additionally, we assessed whether a high level of anti-leukaemia efficacy was maintained when Ara-C was omitted from ATO and ATRA combination therapy.

## Methods

### Eligibility criteria

Eligible patients were those who were less than 14 years old, were newly diagnosed with APL, and had not previously received chemotherapy. A molecular diagnosis was not required for enrollment, but a subsequent molecular confirmation, including the demonstration of PML-RARA transcripts, was required for inclusion in the analysis. A genetic diagnosis was established by detecting the PML-RARA fusion gene using polymerase -chain -reaction (PCR) assays [19, 20] or by demonstrating t (15; 17) translocation using conventional karyotyping or fluorescence in situ hybridization (FISH) [21]. Written informed consent was obtained from all patients before study entry.

### Study design and treatment groups

**Fig. 1:**
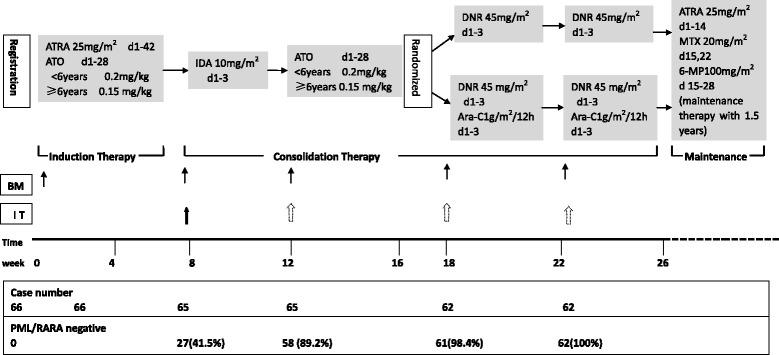
The CCAPL 2010 regimen and MRD test results. BM, bone marrow aspiration; IT, intrathecal injection; ATRA, all-trans-retinoic acid; ATO, arsenic trioxide; DNR, daunorubicin; Ara-C, cytosine arabinoside; MTX, methotrexate; 6-MP, 6-mercaptopurine

The study was a prospective, randomized, single-centre trial. It was designed to determine whether the combination of ATRA and ATO is safe and effective in paediatric APL and whether Ara-C can be omitted when ATO is added for 2 courses. Patients were assigned to receive ATRA plus ATO for induction followed by 1 consolidation course of idarubicin (IDA) and 1 consolidation course of a 28-day cycle of ATO. The patients were then randomly assigned using a computer-generated random allocation schedule to receive 2 courses of either daunorubicin (DNR) or DNR + Ara-C. Patients who were treated with DNR alone were included as the no-Ara-C group. Patients who were treated with DNR + Ara-C were included as the Ara-C group. The patients were subsequently treated with maintenance therapy consisting of oral ATRA, 6-mercaptopurine, and methotrexate for 1.5 years. When CR was achieved, all patients received a prophylactic intrathecal injection (cytarabine, methotrexate, and dexamethasone) for the first time. The patients with an initial white blood cell count > 10 × 10^9^/L then received intrathecal injection once every course. Patients with an indication of CNS leukaemia received intrathecal injection once every other day until normal results were achieved. The regimen is shown in Fig. 1. This trial was conducted in accordance with the Declaration of Helsinki and was retrospectively registered at Clinical- Trials.gov (identifier: NCT01191541).

All children were monitored using reverse transcription polymerase chain reaction (RT-PCR) of bone marrow samples [19]. To amplify the PML/RARa fusion gene, a two-step qualitative RT-PCR analysis was performed as previously described [19]. From January 2011, real-time quantitative PCR (RQ-PCR) was used to identify the PML/RARa fusion transcript [20]. In the RQ-PCR method, established in our laboratory based on cDNA, a dilution of the NB4 cell line reached a sensitivity of 1 × 10^− 5^ for PML-RARa. Bone-marrow morphology, cytogenetics, and RT-PCR/RQ-PCR for PML-RARA were assessed after induction and each consolidation cycle. After consolidation, the patients were assessed every 3 months for 1 year and then every 6 months for 1 year. No pharmaceutical company was involved in the design of the study, data collection or analysis, or the writing of the manuscript.

### Criteria for response and end points

Haematological complete remission (HCR) and haematologic relapse were defined as described in previous publications [1, 9]. Molecular remission was defined as undetectable PML/RARa fusion transcripts. Molecular relapse was defined as the detection of the fusion oncogene PML/RARa in multiple samples within 2 weeks in the same patient. Early death (ED) was considered a death that occurred within two weeks of the beginning of treatment.

The follow-up of the patients was updated in May 2017. The overall survival (OS) durations were calculated as the date of diagnosis to the date of last follow-up or death. Event-free survival (EFS) was defined as the time from diagnosis to the time at last follow-up or an event (i.e., relapse or death). Disease-free survival (DFS) was calculated as the time from the day HCR was achieved to the date of the last follow-up or an event (i.e., relapse). Death at any time and relapse were considered events for the EFS curve, while death in HCR and relapse were considered for DFS curves. Due to the open label character of the study, survival analysis was performed on an intention-to-treat (ITT) and a per-protocol (PP) basis.

### Supportive measures and management of complications

Coagulopathy was treated using fresh frozen plasma or fibrinogen. Platelet transfusions were administered to maintain a platelet count above 50 × 10^9^/L until any significant sign of coagulopathy was resolved. The patients were administered hydroxyurea (1–1.5 g per day), or homoharringtonine (HHT, 1–2 mg per day for 5–10 days) when their peripheral white blood cell (WBC) counts were greater than 25 × 10^9^/L. At the earliest manifestation of suspected differentiation syndrome, ATRA, arsenic trioxide, or both were temporarily discontinued, and intravenous dexamethasone was administered at a dose of 5–10 mg/m^2^ until these signs and symptoms disappeared. Antibiotics and antifungal drugs were administered for fever when required.

### Detection of arsenic concentration

Forty-one patients and 11 healthy children as controls were included in the study to analyse the arsenic concentrations. All samples were collected on Oct 10, 2016. The arsenic concentration in collected plasma, urine, hair, and nail samples was determined using inductively coupled plasma mass spectrometry (ICP-MS). For each assay, 2 mL of plasma, 5 mL of urine or 0.1–0.5 g of nails or hair was collected. Plasma and urine specimens were stored at 4 °C and analysed within 2 weeks. Other specimens were collected in polypropylene tubes. An Agilent 7700× ICP-MS (Agilent technology, USA) equipped with a pure He octopole reaction system (ORS) was used for the total arsenic analysis. No polyatomic interference or argon chloride interference was observed while using this system. The ICP-MS instrument operating conditions are shown in Table 1. A 1.0 mL volume of blood (or urine) or 0.1 g of hair (or nails) was digested in 2 mL of HNO_3_ (65%) and 1 mL of H_2_O_2_ (30%) in a microwave digestion system and then diluted to a total volume of 8 mL using deionized water. (Nitric acid (UP, China), BV-III grade). A blank digest was performed using the same method. All sample solutions were clear. The following digestion conditions were used for the microwave system: 5 min at 1300 W and 160 °C, 5 min at 1300 W and 200 °C, and 20 min at 1300 W and 200 °C. The digested samples were filled to the final volume using ultrapure water and then analysed using ICP-MS. A standard curve was generated for a linear range of 0 to 20 ng/ml and a detection limit of 0.01 μg/L.

**Table 1:** ICP-MS instrument (Agilent 7700×) operating condition

RF power	1550 W
RF matching	1.8 V
Sample depth	10 mm
Carrier gas	1.0 L/min
Nebuliser pump speed	0.1 mL/min
Spray chamber temp	2 °C
Peak pattern	1 point
Reaction cell gas flow	He (4.5 mL/min)
Replicates	3

### Statistical analysis

The primary objective was to demonstrate the noninferiority of DNR alone compared to DNR + Ara-C in terms of the DFS rate at 2 years. Assuming a 95% rate of DFS in the two groups, a margin of − 14%, 5% type 1 error, and 80% power, 31 evaluable patients per group were required to draw a noninferiority conclusion.

The characteristics of all of the included patients were summarized using cross-tabulations (for categorical variables) and quantiles (e.g., the median; for continuous variables). Nonparametric tests were used to analyse comparisons between groups (i.e., χ^2^ and Fisher’s exact tests for categorical variables). EFS, DFS and OS were estimated using the Kaplan -Meier method, and log-rank tests were used for comparisons. All *P* values were two-sided, and those with values of 0.05 or less were considered to be statistically significant. All statistical analyses were performed using SPSS 16.0 software.

## Results

Between May 2010 and December 2016, 66 consecutive paediatric (≤14 years of age) patients who were genetically confirmed with a new diagnosis of APL were admitted in our hospital. The follow-up of the patients was updated in May 2017 and included a median of 36 months (range, 5 to 83 months). One patient ended treatment for economic reasons. The main clinical and biologic characteristics of these patients are shown in Table 2.

**Table 2:** Clinical and Biological Characteristics of the Eligible Patients

Parameter	Total	no-Ara-c group	Ara-c group	*P*
N	66	35	30	
Age, years				
Median	8	8.0	8	0.119
Range	2–14.0	2–14.0	2–13.0	
Gender				0.749
Male	43	22	20	
Female	23	13	10	
WBC count, 10^9^/L				0.220
Median	4.71	5.74	4.23	
Range	0.82–202.4	0.82–202.4	0.99–130	
WBC ≤ 10 × 10^9^/L (n, %)	44 (66.7%)	22 (62.9%)	22 (73.3%)	0.362
WBC > 10 × 10^9^/L (n, %)	22 (33.3%)	13 (37.1%)	8 (26.7%)	
Haemoglobin count, g/L				0.075
Median	79	81.5	78	
Range	44–127	49–127	44–124	
Platelet count, 10^9^/L				0.418
Median	30.5	32.0	24.5	
Range	2–130	6–130	2–125	
PML-RARA				0.846
Long transcript	26	13	12	
Short transcript	17	8	9	
Variable	9	6	4	
Not done	14	8	5	

Fms-like tyrosine kinase 3 (FLT3) mutations were analysed in all patients in total, 11 patients (16.7%) had a FLT3-internal tandem duplication (ITD) mutation and 10 (15.2%) had a FLT3-tyrosine kinase domain (TKD) D835 mutation. There were no significant difference in FLT3-ITD mutation between the patients with WBC > 10 × 10^9^/L and WBC ≤10 × 10^9^/L (*P* = 0.310). C-KIT mutations were identified in 2 (3.0%) patients, a K-RAS mutation was identified in 1 (1.5%) patient, and a TET2 mutation was identified in 1 (1.5%) patient.

### Induction therapy

Among the 66 patients, some had severe symptoms at presentation. These included intracranial bleeding in 4 (6.1%), intraocular bleeding in 6 (9.1%), and mild partial splenic embolization in 2 (3.0%). There were no significant difference in the rate of severe symptoms between the patients with WBC > 10 × 10^9^/L and WBC ≤10 × 10^9^/L (*P* = 0.225). No early deaths occurred. One patient ended treatment for economic reasons. A total of 65 patients were evaluated to determine their response to induction therapy. Haematologic complete remission was achieved in all of these patients.

During induction, hyperleukocytosis (> 10 × 10^9^/L) developed in 59 (90.8%) of the 65 patients with peak WBC counts ranging from 12.8 to 267.8 × 10^9^/L(median, 38.0 × 10^9^/L). In addition, 24 (36.9%) of the 65 patients exhibited an increase in peak WBC counts to more than 50 × 10^9^/L. HHT was used in 28 patients. The dosage of HHT was 1–2 mg/d, and it was administered for 2 to 15 days (median, 7 days). After CR was achieved, 11 (11/28, 39.3%) of the HHT-treated patients tested negative for PML-RARA fusion transcripts, whereas of the patients without HHT, 16 (16/37, 43.2%) tested negative for PML-RARA fusion transcripts. There was no significant difference in the proportion of patients who were negative for PML-RARA fusion transcripts between those who were treated with or without HHT (*P* = 0.749). There was also no significant difference in initial WBC, Hb, and PLT counts and outcomes between the two groups.

During induction therapy, retinoic acid syndrome (RAS) was diagnosed in 9 (13.8%) patients, but it did not contribute to any deaths. Four (6.2%) of the 65 patients suffered Common Terminology Criteria for Adverse Events (CTCAE V.4.0) grade 1–2 hepatotoxicity. Other ATO-associated adverse reactions included extremity oedema in 9 (13.8%) cases, nausea in 2 (3.1%) cases, skin pigmentation in 2 (3.1%) cases, bone ache in 2 (3.1%) cases, cardiac arrhythmia in 1 (1.5%) case and asymptomatic QTc prolongation on electrocardiography in 2 (3.1%) cases. Additional ATRA-associated adverse reactions included headache in 24 (36.9%) cases, skin rash in 3 (4.6%) cases, nausea in 7 (10.8%) cases, abdominal pain in 2 (3.1%) cases, bone ache in 8 (12.3%) cases and skin desquamation in 4 (6.2%) cases. All toxicity events were reversed by appropriate management.

### Consolidation therapy

Consolidation therapy was administered in all patients except for the patient who ended therapy early. No patient died after CR was achieved. Side-effects included sepsis in 6 (9.2%) cases, and hepatotoxicity in 3 (4.6%) cases. No secondary malignancies have so far been reported in our patients.

According to the results of our regimens, which were applied to 2 groups using random selection, 31 patients were included in the Ara-C group, and 34 patients were included in the no-Ara-C group. One patient in the Ara-C group voluntarily transferred to the no-Ara-C group before the random treatment was administered. After the first course of random treatment was administered, three patients in the Ara-C group voluntarily transferred to the no-Ara-C group due to haematologic toxicity. Finally, 30 patients were included in the Ara-C group with 57 courses of treatment and 35 patients in the no-Ara-C group with 73 courses of treatment. There were no significant differences in baseline characteristics between the Ara-C and no-Ara-C groups on a PP basis analysis (Table 2). Also, there was no significant difference (*P* ≥ 0.05) between the two groups on an ITT basis analysis in baseline characteristics (data not shown). In addition, there was no difference in EFS, DFS and OS between the two groups on an ITT and a PP basis analysis. Based on the actual application of the treatment, we compared the hematology toxicity between the two groups. The percentages of courses that included platelet and red blood cell (RBC) transfusions in the Ara-C group were 91.2% (52/57) and 24.6% (14/57), respectively. During consolidation, no blood product was required in the no-Ara-C group. A total of 84.2% (48/57) and 5.5% (4/73) of the patients in the Ara-C and no-Ara-C groups, respectively, had WBC counts <1.0 × 10^9^/L (*P* = 0.000). In the Ara-C group, the median lowest WBC count was 0.62 × 10^9^/L (range, 0.02 to 1.82 × 10^9^/L). The median days of neutropenia was 0 day (range, 0 to 9 days) in the no-Ara-C group and 6 days (range, 0 to 13 days) in the Ara-C group, respectively (*P* = 0.000). There were 6 cases of sepsis, including five in the Ara-C group and one in the no-Ara-C group. No deaths occurred during consolidation therapy.

### MRD tests

Twenty-seven (41.5%) of the 65 patients who were tested after induction were negative for PML-RARA fusion transcripts. After the first consolidation cycle, 58 (89.2%) of the 65 patients were negative, and after the second ATO treatment cycle, 64 (98.5%) of the 65 patients tested negative. After the third consolidation cycle (i.e. the first cycle of DNR/DA), a complete remission (molecular) was achieved in all patients (Fig. 1). There were no significant differences in baseline characteristics between the cohorts that were positive or negative after induction and IDA chemotherapy.

### Prognostic factors and their impact on relapse and survival

Of the 65 patients who entered haematologic CR, the median follow-up time was 36 months (range, 5 to 83 months). Only one patient in the no Ara-C group relapsed. After a median follow-up of 36 months, the EFS was 97.3 ± 2.7%, and the OS was 100%. No factor impacted relapse or survival in our study.

### Arsenic retention on follow-up

Arsenic concentrations were assayed in plasma, urine, hair, and nail samples during and after the cessation of arsenic treatment in forty-one patients. Eleven healthy children were used as the control group. In our patients, 5 ceased ATO treatment after fewer than 3 months, 9 ceased ATO treatment after 3–12 months, 7 ceased ATO treatment after 12–24 months, and 20 ceased ATO treatment after more than 24 months.

**Fig. 2:**
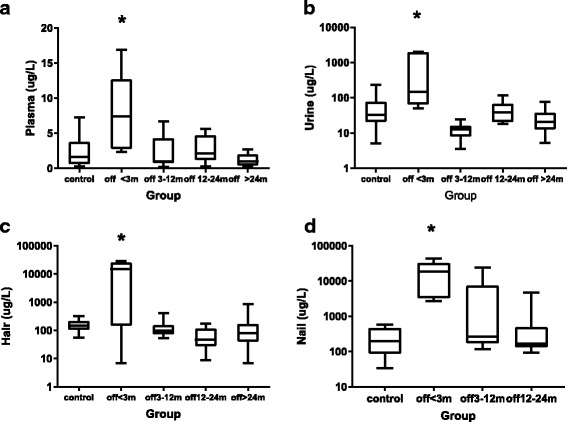
Arsenic concentrations in the plasma (**a**), urine (**b**), hair (**c**) and nail (**d**) samples obtained from the different groups. m, months. * indicates a *P* value less than 0.05 in a comparison with the control group

Figure 2 shows the arsenic concentrations in the plasma, urine, hair, and nail samples at different time points and compared to the control group. Patients who had been off of the arsenic-containing treatment for less than 3 months had higher arsenic concentrations in the plasma, urine, hair and nail samples than the patients in the control group. The arsenic levels in the nails obtained from patients who had ceased treatment for 3–12 months (median, 264.2 ng/g; range, 117–24,240 ng/g) were higher than the levels in the controls (median, 198.8 ng/g; range, 33.6–588.1 ng/g). But statistical analyses revealed no significant difference between the two groups (*P* = 0.215). There was no difference in the median arsenic concentrations in plasma, urine, hair, and nail samples between patients in whom arsenic treatment had been ceased for more than 12 months and normal controls.

## Discussion

Because earlier findings showed that ATO, with or without ATRA was highly effective in adult APL patients and allowed the intensity of chemotherapy to be minimized, we performed a clinical trial in 2010 to determine whether ATO plus ATRA was safe and effective in children and whether we could eliminate Ara-C therapy from consolidation when ATO was added for 2 courses.

ATRA-ATO was recently shown to have an advantage over ATRA-chemotherapy in large, randomized adult trials. This option has since become the new standard of care for low-risk patients [5, 7, 8, 10, 22]. The APL0406 randomized trial showed that in patients with non-high-risk APL, better outcomes were achieved in those treated with ATRA-ATO than in those treated with standard ATRA-chemotherapy [7, 8]. The long term follow-up results of this trial are especially supportive of the advantages of ATRA-ATO over ATRA-chemotherapy, showing that they increase over time [22]. Studies in children with APL showed minimal toxicities and favourable outcomes when they were treated with the ATO and ATRA combination during induction [12, 14]. Creutzig U et al. [13] also reported results for paediatric APL patients treated with the ATRA-ATO regimen that resembled the Lo-coco regimen for adults [7]. No patient died early after diagnosis or during induction. Treatment with ATRA and ATO was well tolerated in their paediatric standard- risk patients with APL [13]. In our CCAPL2010 study, no patients had an early death, and only one patient relapsed. All toxicity events were tolerable, and all were reversed by appropriate management. All of our patients achieved molecular complete remission after the third consolidation cycle. The results of our study indicated that our protocol, which included ATO, ATRA and cytotoxic chemotherapy, achieved good outcomes with only moderate side-effects in children.

In the pre-ATO era, the role of Ara-C in APL was controversial [15–17]. However, the role of Ara-C when a combination ATRA and ATO treatment was applied for paediatric APL had not been studied as of 2010. In our study, there was no difference in outcomes between the Ara-C group and the no Ara-C group on an ITT and a PP basis analyses. These results indicated that in paediatric APL, Ara-C can be omitted, at least when regimens similar to ours are applied.

It has been suggested that higher cumulative doses of anthracyclines yield better results in APL [23]. However, a higher cumulative dose may also lead to cardiac toxicity, especially in children [24]. In our study, the cumulative dose of anthracycline was 420 mg/m^2^. To date, no severe anthracycline-related cardiac toxicity has occurred. However, cardiac complication from anthracyclines should be monitored every year after completion of therapy. Recent results have shown that the ATRA+ATO combination (without chemotherapy) is at least as effective as the classical ATRA+CT regimens in low –risk APL patients and is also less myelosuppressive [7]. However, when our CCAPL2010 trial began, the feasibility of treatment of APL without chemotherapy was unknown. Thus, our patients with low- risk APL may have been over-treated. With increasing evidence confirming the efficacy of therapy including ATO, a chemotherapy-sparing approach with ATO should be planned for paediatric APL low-risk patients. Additionally, suitable treatment for paediatric patients with high-risk APL should be further studied.

In the pre-ATO era, the prognostic factors for APL included the presenting WBC and platelet counts, gender, CD56 expression, HLA-B13, and the subtype of fusion product [25]. WBC and platelet counts are especially considered the definitive indexes for relapse risk [26]. However, in the ATO era, the prognostic value of many of these factors has been questioned [27, 28]. Lou et al. [27] examined records from 184 APL patients who were treated with ATRA+ATO and found that there was no association between the 3-year relapse-free survival (RFS) rate and presenting WBC counts, FLT3-ITD status, or PML/RARA isoforms. In line with previous ATO-based upfront studies, we did not identify any prognostic indicators in our study.

ATO has been shown to be the most potent single agent in APL therapy [3, 4]. However, an increase in the risk of solid cancers has been reported in patients with long-term exposure to low doses of inorganic arsenic compounds [29]. Fortunately, Zhou J et al. [4] reported that no severe side-effects were documented in patients who continued ATO therapy for more than 3 years, nor were second malignancies encountered after a follow-up of 3 or more years after the completion of therapy. Our patients received ATO for 56 days. The side-effects of ATO were moderate and reversible given appropriate management. An analysis of arsenic levels in the plasma, urine, nails and hair of patients indicated that there was no significant accumulation of arsenic after ATO had been discontinued for 12 months. Further investigations that include long-term follow up times are needed.

Notably, no early deaths occurred in our study. Some patients died of intracranial haemorrhage in transit, which might provide an explanation. Although 4 (6.1%) patients with intracranial haemorrhage were admitted to our hospital, none of the patients died. Early recognition of APL, prompt ATRA/ATO administration and aggressive supportive care might explain this outcome.

## Conclusion

The results of our study indicate that ATO is safe and effective in paediatric APL and that Ara-C can be omitted, at least when using regimens similar to ours. Unfortunately, our patients with low- risk APL may have been over-treated. A decreased intensity of treatment in paediatric APL patients should be further studied based on the ATRA and ATO combination.

## Abbreviations

APL: Acute promyelocytic leukemia
ATO: Arsenic trioxide
ATRA: All-trans-retinoic acid
CR: Complete remission
DFS: Disease-free survival
ED: Early death
EFS: Event-free survival
FISH: Fluorescence in situ hybridization
FLT3: Fms-like tyrosine kinase 3
HCR: Haematological complete remission
ICP-MS: Inductively coupled plasma mass spectrometry
ITD: Internal tandem duplication
ORS: Octopole reaction system
OS: Overall survival
PCR: Polymerase-chain-reaction
RAS: Retinoic acid syndrome
RBC: Red blood cell
RFS: Relapse-free survival
TKD: Tyrosine kinase domain
WBC: White blood cell
